# Demand-side financing for maternal and newborn health: what do we know about factors that affect implementation of cash transfers and voucher programmes?

**DOI:** 10.1186/s12884-017-1445-y

**Published:** 2017-08-31

**Authors:** Benjamin M. Hunter, Susan F. Murray

**Affiliations:** King’s College London, Department of International Development, The Strand, London, WC2R 2LS UK

**Keywords:** Demand-side financing, Vouchers, Cash transfers, Maternal health, Newborn health, Implementation

## Abstract

**Background:**

Demand-side financing (DSF) interventions, including cash transfers and vouchers, have been introduced to promote maternal and newborn health in a range of low- and middle-income countries. These interventions vary in design but have typically been used to increase health service utilisation by offsetting some financial costs for users, or increasing household income and incentivising ‘healthy behaviours’. This article documents experiences and implementation factors associated with use of DSF in maternal and newborn health.

**Methods:**

A secondary analysis (using an adapted Supporting the Use of Research Evidence framework – SURE) was performed on studies that had previously been identified in a systematic review of evidence on DSF interventions in maternal and newborn health.

**Results:**

The article draws on findings from 49 quantitative and 49 qualitative studies. The studies give insights on difficulties with exclusion of migrants, young and multiparous women, with demands for informal fees at facilities, and with challenges maintaining quality of care under increasing demand. Schemes experienced difficulties if communities faced long distances to reach participating facilities and poor access to transport, and where there was inadequate health infrastructure and human resources, shortages of medicines and problems with corruption. Studies that documented improved care-seeking indicated the importance of adequate programme scope (in terms of programme eligibility, size and timing of payments and voucher entitlements) to address the issue of concern, concurrent investments in supply-side capacity to sustain and/or improve quality of care, and awareness generation using community-based workers, leaders and women’s groups.

**Conclusions:**

Evaluations spanning more than 15 years of implementation of DSF programmes reveal a complex picture of experiences that reflect the importance of financial and other social, geographical and health systems factors as barriers to accessing care. Careful design of DSF programmes as part of broader maternal and newborn health initiatives would need to take into account these barriers, the behaviours of staff and the quality of care in health facilities. Research is still needed on the policy context for DSF schemes in order to understand how they become sustainable and where they fit, or do not fit, with plans to achieve equitable universal health coverage.

## Background

Women and their families can face multiple barriers to accessing maternity care services, and financial barriers are a well-documented concern [1, 2]. In many countries there are demands for out-of-pocket formal fees and informal payments for care services or supplies such as medicines, sutures, gloves and diagnostic tests [3, 4]. There are the practical and financial difficulties of travel to health centres and the opportunity costs of being away from home or from work, or from dependents. For poor families, any such costs can cause severe financial hardship [5], and may result in delays or avoidance of care-seeking that increase health risks for mothers and newborns and further escalate costs. One approach to ameliorate these effects is the use of ‘demand-side’ financing (DSF) interventions that are designed to promote health by providing cash or vouchers to users to offset some of the financial costs of using or getting to maternity care services, or to increase household income and incentivise ‘healthy behaviours’.

Within this overarching definition there are five types of DSF that have been used in the health sector and there are important differences in their intended mechanism of action [6, 7]. *Conditional cash transfers*, which have been widely used in Latin America, aim to increase utilisation of maternity care services by making regular payments to households linked to ‘conditionalities’ including attendance at community meetings and uptake of government health services. These are primarily focused on child health and development but some schemes include maternity care uptake. *Unconditional cash transfers* are similar regular payments but, in the absence of specific conditionalities for service utilisation, have the more general aim of alleviating the effect of poverty on a woman’s health during pregnancy. *Short-term cash payments to offset costs* are typically retrospective payments made at government health facilities to those who attend for care. *Vouchers for maternity care services* aim to reduce the cost of maternity care services and *vouchers for ‘merit’ goods* aim to reduce the cost of merit goods (such as food or insecticide-treated nets) that promote maternal health. Vouchers may be distributed by community-based workers or at health facilities, and voucher schemes may be designed to incorporate services in the private sector as well as government facilities.

There have been seven systematic reviews of evidence on the impact of DSF mechanisms on maternal health during the period 2007–2012 [7–13]. Two of the reviews examined the impact of cash transfers [9, 11], three the impact of vouchers [8, 12, 13], and two the role of multiple types of DSF [7, 10]. The systematic reviews reported on a rapidly growing body of evidence that DSF can lead to a short-term increase in uptake of maternity care services, but could offer little evidence on longer-term effects on service uptake or maternal and neonatal morbidity and mortality. A recently published systematic review has confirmed these findings [14].

This article has been commissioned by the World Health Organization’s Department of Maternal, Newborn, Child and Adolescent Health as part of a series of articles on health promotion interventions. The series aims to document factors that affect programme implementation in order to support policy-makers and decisions on how best to improve access to skilled care during pregnancy, childbirth and after birth. This article focuses on factors that have been found to affect implementation of DSF programmes in maternal and newborn health and has three components: a review of stakeholder perspectives and experiences of DSF interventions; information on the barriers and facilitators to implementation of the interventions, and a discussion on how these relate to the improvements in care-seeking outcomes reported elsewhere and what this means for programmatic initiatives [14].

## Methods

This article is a secondary analysis of studies identified in a systematic review that was conducted in 2012 [6, 7] and repeated in 2015 [14]. The systematic review used the Joanna Briggs Institute approach, which incorporates both quantitative and qualitative data into reviews and has been used to review evidence on a range of policy and healthcare systems topics [15]. The population of interest in the review was economically poor women who were pregnant or within 42 days of end of pregnancy, in the context of low- and middle-income (both lower- and upper-middle) countries as defined by the World Bank at the time the study was published. The intervention of interest was DSF as a mechanism to increase consumption of goods and services intended to impact positively on maternal and newborn health. Outcomes of interest in the systematic review related to the effectiveness of DSF programmes to promote uptake of maternity care services and maternal and newborn health, and wider impact on quality of care. In addition to questions of effectiveness, the 2012 review also analysed qualitative research relating to barriers and facilitators for effective and sustainable programme implementation.

The systematic searches for the review used 30 terms in 19 medical, health and social policy databases and seven databases of unpublished research, and aimed to retrieve quantitative and qualitative studies that were published between January 1990 and June 2015. Retrieved studies were examined using Joanna Briggs Institute tools for critical appraisal of quantitative and qualitative research that include questions on study methods and the presentation of findings [15]. The review team assigned an overall quality rating to individual studies using a three-point rating system (low-, medium- or high-quality), similar to that used to assess study bias in the Effective Public Health Practice Project (EPHPP) quality assessment tool. The rating assigned to each study was based on assessments of study methods and reporting using critical appraisal tools produced by the Joanna Briggs Institute. The assessments are described in detail in a linked systematic review [14].

Data were extracted from included studies using standardised tools developed by the Joanna Briggs Institute, and the findings presented in this article have been analysed thematically using a comprehensive framework for factors affecting implementation of health promotion interventions, adapted from the SURE (Supporting the Use of Research Evidence) framework for preparing policy briefs [16]. The analytical framework, which is described in detail in a paper by Smith et al. in this series, includes five ‘levels’ of factors that affect policy implementation (main stakeholders in communities, healthcare providers, other stakeholders, health service delivery factors and social and political factors) and provides a list of types of barrier and enabler for each level. One of the authors, BMH, read through all included studies and extracted data relating to each level of the framework, then both authors examined the extracted data and re-organised them into themes based on the list of types of barrier and enabler.

## Results

### Range and quality of the body of literature

The article includes findings from 49 quantitative studies and from an additional 49 qualitative studies that contained information relevant to the quantitative studies (see Table 1 for details of included studies) that relate to the five types of DSF in 22 country programmes:

**Table 1:** Table of characteristics of included studies

Study	Study design	Setting	Description of intervention	Recommendation	Outcomes of interest reported
Conditional cash transfers					
*Oportunidades, Mexico*					
Barber and Gertler (2008)	Cross-sectional	Rural communities, Mexico	Oportunidades (conditional cash transfers)The programme was launched in 1997 as PROGRESA and later renamed Oportunidades. Monthly cash transfers are paid directly to mothers and are conditional on meeting health and education requirements. These included regular antenatal visits for pregnant women and attendance at health education meetings.	Conditional cash transfers	Perinatal morbidity, ANC
Barber and Gertler (2009)	Cross-sectional	Rural communities, Mexico		Conditional cash transfers	QoC
Barber (2010)	Cross-sectional	Rural communities, Mexico		Conditional cash transfers	C/I
Barham (2011)	Retrospective area study	Rural communities, Mexico		Programme less effective in areas that do not meet minimum level of sanitation	Infant mortality, NM
Hernandez Prado et al. (2004)	Retrospective area study	Rural, semi-urban and urban areas, Mexico		Conditional cash transfers	MM, infant mortality
Hernandez Prado et al. (2004)	Repeat cross-sectional	Rural, semi-urban and urban areas, Mexico		Conditional cash transfers	ANC, SBA, C/I, QoC, infant morbidity
Sosa-Rubai et al. (2011)	Cross-sectional	Rural communities, Mexico		Conditional cash transfers, but need better targeting to marginalised groups	ANC
Urquieta et al. (2009)	Repeat cross-sectional	Rural communities, Mexico		Conditional cash transfers, but need to include household members who influence decision-making on place of birth	SBA
*Program Keluarga Harapan, Indonesia*					
Alatas et al. (2011)	Repeat cross-sectional	Rural and urban districts in Indonesia	Program Keluarga Harapan (conditional cash transfers)The programme was introduced in 2007 and was targeted to five provinces. It was piloted in sub-districts that were felt had sufficient supply-side capacity to meet additional demand for services. Eligible households included those with pregnant or lactating women and women were expected to attend ANC, use a SBA and receive PN/PP. A cash transfer is paid quarterly to the women through a nearby post office. Facilitators are expected to verify that conditionalities are met.	Conditional cash transfers, but relax conditionalities in areas with weaker health systems	ANC, SBA, FB, PN, NM, infant mortality
Febriany et al. (2011)	Qualitative – interviews and focus groups	12 villages (mixed urban and rural) in 2 provinces, Indonesia		Conditional cash transfers, but need to reduce gaps in service provision and overcome social, economic and geographical barriers	Implementation
Triyana (2012)	Repeat cross-sectional	Rural and urban areas in Indonesia		Conditional cash transfers	MM, NM, infant mortality, SBA, QoC
*Plan de Atención Nacional a la Emergencia Social (PANES), Uruguay*					
Amarante et al. (2011)	Repeat cross-sectional	Uruguay	Plan de Atención Nacional a la Emergencia Social (PANES) (conditional cash transfers)Between April 2005 and December 2007, Plan de Atención Nacional a la Emergencia Social was implemented by the Uruguayan government. Monthly payments were made to eligible households. In homes with a pregnant woman, payments were conditional on her attendance at ANC.	Conditional cash transfers	Perinatal morbidity, maternal morbidity, ANC, SBA
*Muthulakshmi Reddy Maternity Benefit Scheme, India*					
Bala-subramanian and Ravindran (2012)	Cross-sectional	Tamil Nadu, India	Muthulakshmi Reddy Maternity Benefit Scheme (conditional cash transfers)The programme was launched in Tamil Nadu, India, in 1987 and provided unconditional payments (in two instalments) to pregnant women. Payments were increased in size over time and were made conditional on antenatal care attendance in 2012. The eligibility of women is determined by a village health nurse and includes those who meet criteria for poverty and who had previously had no more than two live births.	Conditional cash transfers, but shorter application process needed	FB
Public Health Resource Network (2010)	Qualitative – in-depth interviews	2 districts in Tamil Nadu, India		Conditional cash transfers, but with universal eligibility, timely payments, assistance from community-based workers, and supply-side investment so that maternity care services are universally available and are free	Implementation
*Comunidades Solidarias Rurales, El Salvador*					
De Brauw et al. (2011)	Repeat cross-sectional	Rural communities, El Salvador	Comunidades Solidarias Rurales (conditional cash transfers)The programme was introduced in 2005 and eligible households are identified from census data. Pregnant woman in eligible households must attend ANC and monthly local health education meetings are held. The programme coincided with health system strengthening programmes introduced by the government.	Conditional cash transfers, but should be conditional on ANC during first trimester, SBA and PNC	ANC, FB, SBA, PN
*Bolsa Familia, Brazil*					
Guanais (2013)	Retrospective area study	Brazil	Bolsa Familia (conditional cash transfers)Since 2003 Brazil has paid monthly cash transfers to eligible households that meet conditionalities including that pregnant women attend ANC. Payments are made to women and are credited to electronic benefit cards.	Conditional cash transfers, but should be accompanied by supply-side interventions	Post-neonatal mortality
Shei (2013)	Retrospective area study	Brazil		Conditional cash transfer effective but need to meet increased demand for services and need to target vulnerable groups	NM, post-neonatal mortality, infant mortality
*Mi Familia Progresa, Guatemala*					
Gutierrez et al. (2011)	Repeat cross-sectional	Guatemala	Mi Familia Progresa (conditional cash transfers)Introduced by the Guatemalan government in 2008, Mi Familia Progresa payments are made to eligible households with a pregnant woman and are conditional on ANC attendance.	Conditional cash transfers	ANC, FB, PM
*Programa de Asignación Familia, Honduras*					
Morris et al. (2004)	Repeat cross-sectional	Rural municipalities, Honduras	Programa de Asignación Familia (conditional cash transfers)Vouchers with a cash value were distributed to eligible households on a regular basis. Distribution to households with a pregnant woman was conditional on her ANC attendance.	Conditional cash transfers	ANC, PN
Unconditional cash transfers					
*Child Grant Programme, Zambia*					
Handa et al. (2015)	Repeat cross-sectional	Three rural districts in Zambia	Child Grant Programme (conditional cash transfers)Launched in 2010, the programme operates in three districts with the highest rates of mortality and morbidity. Eligibility is universal within programme areas as long as there is a young child in the household. Payments are made directly to mothers.	Unconditional cash transfers, but need access to maternity services and may need complimentary short-term payments that are conditional on uptake of maternity care services	ANC, QoC, SBA
Short term cash payments to offset costs of access					
*Janani Suraksha Yojana, India*					
Amudhan et al. (2013)	Quasi-experimental pre- and post- comparative with control groups	Rural area in Haryana, India	Janani Suraksha Yojana (payments to offset costs of access)The programme was launched in 2005 as part of the National Rural Health Mission. A cadre of community-based workers (accredited social health activists) were created to promote the programme in communities. Women from low-income households could receive a cash payment if they give birth in a health facility (usually government, although some states also accredited private facilities). Accredited social health activists are salaried and expected to encourage ANC, facility-based births and PN/PP. They are eligible to receive a payment for accompanying a woman to a health facility to give birth.	Short-term payments, but followed by supply-side strengthening	FB
Carvalho et al. (2014)	Cross-sectional	National sample of districts (mixed urban and rural), India		Short-term payments, but need appropriate systems for payments	PN, PP
Chaturvedi and Randive (2009)	Qualitative – semi-structured interviews and focus groups	Ahmednagar district in Maharashtra, India		Short-term payments, but ensure that private service providers are monitored and regulated	Implementation
Chaturvedi and Randive (2011)	Qualitative – semi-structured interviews and focus groups			Ensure sufficient government capacity to design and manage public-private partnerships	Implementation
Chaturvedi et al. (2015a)	Qualitative – observations and interviews	11 health facilities in Madhya Pradesh		Improve quality of care before introducing short-term payment programmes	Implementation
Chaturvedi et al. (2015b)	Qualitative – record reviews and interviews	73 health facilities in Madhya Pradesh		Train and support staff to use partographs	Implementation
Coffey (2014)	Qualitative – interviews and observations	Three villages in a rural district in Uttar Pradesh		Short-term payments, but need to incentivise health outcomes	Implementation
Dasgupta (2007)	Qualitative – maternal death investigations	7 districts in Uttar Pradesh, India		Invest in supply-side capacity, promote awareness of programme benefits, and develop systems to track each pregnancy	Implementation
Devadasan et al. (2008)	Qualitative - interviews	One district in each of four states, India		Short-term payments, but need to ensure quality of care, use streamlined processes for distribution of payments and monitor the programme	Implementation
Gopalan et al. (2012)	Qualitative – interviews and focus groups	Three districts in Orissa		Short-term payments, but with greater protection for families from costs of care	Implementation
Gupta (2007)	Qualitative – interviews and focus groups	Nalanda and West Champaran (rural) districts in Bihar, India		No recommendations made	Implementation
Hangmi and Kuki (2009)	Qualitative – interviews and focus groups	Churachandpur (rural) district in Manipur, India		Short-term payments, but need to invest in capacity of service providers, ensure fair selection of community-based workers, and streamline cash payments	Implementation
Human Rights Watch (2009)	Qualitative – individual and group interviews	Rural areas in Uttar Pradesh, India		Develop monitoring systems	Implementation
Joshi and Sivaram (2014)	Repeat cross-sectional	National sample of districts (mixed urban and rural), India		Concurrent supply-side strengthening and flexibility in guidelines	ANC, SBA, PN
Khan et al. (2010)	Qualitative – interviews	24 villages in Uttar Pradesh (rural), India		Short-term payments, but with adequate training and incentives for community-based workers, and inclusion of private service providers	Implementation
Krishna and Ananthpur (2011)	Qualitative – interviews and focus groups	Gulbarga and Raichur (rural) districts in Karnataka, India		No recommendations made	Implementation
Kumar et al. (2009)	Qualitative – focus groups	Una (predominantly rural) district in Himachal Pradesh, India		Short-term payments, but with streamlined distribution of cash payments and ensure community-based workers appropriately trained and that posts are filled	Implementation
Lim et al. (2010)	Repeat cross-sectional	National sample of districts (mixed urban and rural), India		Short-term payments, but need improved targeting and quality of care	ANC, FB, SBA, PM, NM
Lodh et al. (2009)	Qualitative – interviews and focus groups	Muzaffarpur district (predominantly rural) in Bihar, India		Short-term payments, but with streamlined distribution of cash payments	Implementation
Mazumdar et al. (2012)	Repeat cross-sectional	National sample of districts (mixed urban and rural), India		Short-term payments, but caution regarding unintended consequences	ANC, FB, SBA, C/I, NM
Nandan et al. (2008)	Qualitative – semi-structured and in-depth interviews and focus groups	3 districts in Orissa (predominantly rural), India		Short-term payments using streamlined payment systems, inclusion of private service providers, supply-side investment and awareness generation	Implementation
Nandan et al. (2008)	Qualitative – semi-structured and in-depth interviews and focus groups	3 districts in Orissa, India		Short-term payments using streamlined payment systems, supply-side investment and awareness generation	Implementation
Purohit et al. (2014)	Cross-sectional	Four districts in Rajasthan, India		Short-term payments	ANC, PN, QoC
Rai et al. (2012)	Qualitative – in-depth interviews and focus groups	12 villages in Jharkhand, India		Short-term payments alongside investment in supply-side capacity	Implementation
Randive et al. (2013)	Repeat cross-sectional	284 districts across nine Indian states		Short-term payments, but need to improve quality of care to impact on MM	FB
Santhya et al. (2011a)	Repeat cross-sectional	Alwar and Jodhpur districts (mixed rural and urban), India		Short-term payments, but improve awareness among communties, quality of services and administrative capacity	ANC, FB, SBA, PP, QoC, oxytocin
Santhya et al. (2011b)	Qualitative – in-depth interviews	Alwar and Jodhpur districts in Rajasthan, India		Short-term payments with trained community-based workers to raise awareness in communities	Implementation
Singh and Chaturvedi (2007)	Qualitative – focus groups and interviews	Five districts in Uttar Pradesh and three districts in Uttarakhand, India		Short-term payments using streamlined systems, inclusion of postpartum care and removal of formal and informal user fees	Implementation
Sri et al. (2012)	Qualitative – maternal death reviews	Barwani district, Uttar Pradesh, India		Investments must be made in supply-side strengthening and ensuring quality of care before short-term payments are considered	Implementation
Uttekar et al. (2007a)	Qualitative - interviews	3 districts in Himachal Pradesh, India		Short-term payments, but need to ensure quality of care by investing in supply-side capacity, use streamlined payments and raise awareness among hard-to-reach communities	Implementation
Uttekar et al. (2007b)	Qualitative - interviews	3 districts in West Bengal, India		Short-term payments, but need to ensure quality of care by investing in supply-side capacity, use streamlined payments and raise awareness among hard-to-reach communities	Implementation
Uttekar et al. (2007c)	Qualitative - interviews	3 districts in Orissa, India		Short-term payments, but need to ensure quality of care by investing in supply-side capacity, use streamlined payments and raise awareness among hard-to-reach communities	Implementation
Uttekar et al. (2007d)	Qualitative - interviews	3 districts in Rajasthan, India		Short-term payments, but need to ensure quality of care by investing in supply-side capacity, use streamlined payments and raise awareness among hard-to-reach communities	Implementation
Uttekar et al. (2007e)	Qualitative - interviews	3 districts in Assam, India		Short-term payments, but need to ensure quality of care by investing in supply-side capacity, use streamlined payments and raise awareness among hard-to-reach communities	Implementation
Uttekar et al. (2008a)	Qualitative - interviews	3 districts in Uttar Pradesh, India		Short-term payments, but need to ensure quality of care by investing in supply-side capacity, use streamlined payments and raise awareness among hard-to-reach communities	Implementation
Uttekar et al. (2008b)	Qualitative - interviews	3 districts in Bihar, India		Short-term payments, but need to ensure quality of care by investing in supply-side capacity, use streamlined payments and raise awareness among hard-to-reach communities	Implementation
Vora et al. (2012)	Cross-sectional	Rural areas in Gujarat and Tamil Nadu, India		Short-term payments, but should expand availability of health facilities, develop referral networks, include public and/or private facilities depending on local health system, promote awareness generation, and divide payments across maternity care services to incentivise multiple services.	ANC, FB, C/I
*CHIMACA project, China*					
Hemminki et al. (2013)	Cross-sectional	One county in Anhui province, China	CHIMACA project (payments to offset costs of access)Women could claim a small cash payment from a health centre if they attended ANC between 2007 and 2009. The size of the payment was increased with the number of ANC visits. Village family planning workers distributed leaflets in communities to advertise the programme and midwives distributed the leaflets to women attending ANC.	Short-term payments, but need payments that are appropriately sized and relatively easy to obtain	ANC, C/I, PN, QoC
*SURE-P, Nigeria*					
Okoli et al. (2014)	Retrospective area study	Nine states, Nigeria	SURE-P (short term payments to offset costs of access)Cash payments were made to women who attended specific maternity care services (ANC, childbirth and PN/PP). Eligibilty was geographic and was based on the selection of health facilities participating in the wider SURE-P health programme. If a woman was referred to a higher level hospital, the hospital received a payment.	Short-term payments, but need to improve retention of programme recipients throughout pregnancy	ANC, SBA
*Safe Delivery Incentive Programme, Nepal*					
Powell-Jackson et al. (2009)	Retrospective area study	Makwanpur district (rural), Nepal	Safe Delivery Incentive Programme (payments to offset costs of access)The programme was launched in 2005 and provides a cash payment to women who give birth in a health facility, and to a health worker who attends her birth (either at home or in a health facility). Payments were initially only available to women who have had two or fewer previous live births, but this condition was later removed. The programme was initially limited to government health facilities but later expanded to include the private sector.	Short-term payments, but need to improve quality of care to impact on NM and need to ensure good communication to communities	ANC, FB, SBA, C/I, NM
Powell-Jackson et al. (2009)	Qualitative – interviews and focus groups	10 districts, Nepal		Attention to implementation challenges in countries with poor capacity to administer programmes and provide services, and careful planning	Implementation
Powell-Jackson and Hanson (2012)	Cross-sectional	National sample of districts (mixed urban and rural), India		Short-term payments, but need effective implementation	SBA, FB, C/I
Vouchers for maternity care services					
*Pilot programmes, Pakistan*					
Agha (2011a)	Repeat cross-sectional	Dera Ghazi Khan City (urban), Pakistan	Pilot voucher scheme (vouchers for maternal health services)12 month programme in which vouchers were sold to eligible families and local officials verified eligibility. Vouchers could be exchanged at participating Greenstar facilities for ANC, childbirth (including caesarean section if needed), PP and PN. Providers were reimbursed for each voucher accepted.	Vouchers for maternity care services	ANC, FB, PN
Agha (2011b)	Repeat cross-sectional	Jhang district (rural), Pakistan		Vouchers for maternity care services	ANC, FB, PN
*Maternal Health Voucher Scheme, Bangladesh*					
Ahmed and Khan (2011)	Cross-sectional	Sarishabari district (predominantly rural), Bangladesh	Maternal Health Voucher Scheme (vouchers for maternal health services) Since 2007 poor pregnant women received vouchers (some districts had universal distribution, some had targeted distribution) entitling them to free maternity care services (ANC, childbirth, PN/PP), transport subsidies, a cash incentive for delivery with a qualified provider (at home or in a designated facility) and a gift box. Providers received incentives to distribute vouchers and provide services that were covered by the vouchers.	Vouchers for maternity care services	ANC, FB, SBA, PN
Ahmed and Khan (2011)	Qualitative – semi-structured interviews	Sarishabari district (predominantly rural), Bangladesh		Vouchers for maternity care services, but with significant expansion of service delivery capacity of health facilities	Implementation
Hatt et al. (2010)	Cross-sectional	Early implementation subdistricts (mainly rural), Bangladesh		Vouchers for maternity care services	ANC, FB, C/I, PN
	Qualitative – interviews and focus groups	Early implementation subdistricts (mainly rural), Bangladesh		Vouchers for maternity care services	Implementation
Koehlmoos et al. (2008)	Qualitative – structured and in-depth interviews and focus groups			Vouchers for maternity care services, but with investment in physical infrastructure at government facilities, appropriate human resources, and attention to incentives to conduct caesarean sections	Implementation
Nguyen et al. (2012)	Cross-sectional	Early implementation subdistricts (mainly rural), Bangladesh		Vouchers for maternity care services as part of a wider initiative that includes supply-side strengthening	ANC, FB, SBA, C/I, PN
Reproductive Health Vouchers Evaluation Team (2011)	Qualitative - interviews	22 sub-districts, Bangladesh		Vouchers for maternity care services	Implementation
*Pilot voucher programme, Bangladesh*					
Rob et al. (2009)	Repeat cross-sectional	Habiganj district (rural), Bangladesh	Pilot voucher scheme (vouchers for maternal health services)12 month programme in which fieldworkers identified a total of eligible women who were then validated as “poor” by a community support group Women could use vouchers for ANC, childbirth, PN and PP. Providers were reimbursed for vouchers accepted. Service providers and fieldworkers were trained and strengthened health facilities for providing ANC, delivery, and PNC services.	Vouchers for maternity care services	ANC, FB, SBA, PN
	Qualitative – semi-structured interviews	Habiganj district (rural), Bangladesh		Vouchers for maternity care services	Implementation
*Makerere University Voucher Scheme, Uganda*					
Alfonso et al. (2015)	Retrospective area study	Kamuli and Pallisa districts, Uganda	Makerere University Voucher Scheme (vouchers for maternal health services)Between June 2010 and May 2011, vouchers were distributed to women in two selected areas of two Ugandan districts during ANC visits. Vouchers could be exchanged at public facilities or participating private facilities for intrapartum care, transport to and from the hospital, and for PN/PP in case of complications. Transport vouchers’ reimbursement covered the cost of the average distance in the treatment areas, and health facilities were reimbursed for services provided.	Vouchers for maternity care services	FB
Pariyo et al. (2011)	Qualitative – focus groups	Kamuli district, Uganda		Vouchers for maternity care services, but with inclusion of transport providers	Implementation
*HealthyBaby vouchers, Uganda*					
Okal et al. (2013)	Qualitative - interviews	7 districts, Uganda	HealthyBaby vouchers (vouchers for maternal health services)Since 2008 vouchers for maternity care services have been sold to eligible women in 20 districts. Vouchers can be exchanged at accredited private providers for ANC, childbirth, PN. Community-based voucher distributors are responsible for targeting poor pregnant women using district-customized poverty grading tool.	Vouchers for maternity care services, but with public facilities included, greater awareness raising in communities and support for policy champions	Implementation
Reproductive Health Vouchers Evaluation Team (2012)	Repeat cross-sectional	6 districts, Uganda		Vouchers for maternity care services	ANC, FB, PN
	Qualitative - interviews	30 health facilities, Uganda		Vouchers for maternity care services	Implementation
*Vouchers for Health, Kenya*					
Abuya et al. (2012)	Qualitative – in-depth interviews	Three districts and two urban slums	Vouchers for Health (vouchers for maternal health services)Since 2006 vouchers have been sold to eligible women in three districts and two informal settlements in Kenya. Vouchers can be exchanged for ANC, childbirth (including caesarean section if needed) and PN. A facility was accredited if it met criteria set by the public authorities in terms of staffing and quality of care.	Vouchers for maternity care services, with strong partnerships between public and private sectors and leading role for government	Implementation
Amendah et al. (2013)	Repeat cross-sectional	Two urban slums in Nairobi, Kenya		Vouchers for maternity care services	FB for subsequent pregnancy
Arur et al. (2009)	Qualitative – semi-structured interviews	Kenya		Vouchers for maternity care services if financial issues are the main barrier to care-seeking	Implementation
Bellows et al. (2012)	Repeat cross-sectional	Informal settlements in Nairobi (urban), Kenya		Vouchers for maternity care services	ANC, FB, SBA
Njuki et al. (2013)	Qualitative – structured interviews and focus groups	Three districts in Kenya		Vouchers for maternity care services, but with adequate information and availability of voucher distributors	Implementation
Njuki et al. (2015)	Qualitative – in-depth interviews	Three districts in Kenya		Vouchers for maternity care services, but greater awareness of eligibility criteria and entitlements, and greater flexibility for public facilities to use income from vouchers	Implementation
Obare et al. (2012)	Cross-sectional	Six districts (all mixed urban and rural), Kenya		Vouchers for maternity care services	ANC, FB, SBA, PN
Obare et al. (2014)	Repeat cross-sectional	Six districts (all mixed urban and rural), Kenya		Vouchers for maternity care services, but transport costs should be included	FB
Reproductive Health Vouchers Evaluation Team (2011)	Qualitative - interviews	6 rural districts and 2 informal settlements, Kenya		Vouchers for maternity care services with awareness generation, monitoring of quality of care, and adequate provisions to overcome geographical (transports) issues	Implementation
Watt et al. (2015)	Repeat cross-sectional	Six districts (all mixed urban and rural), Kenya		Vouchers for maternity care services, and need to include PP before discharge from hospital within hospital agreements	QoC
*Chiranjeevi Yojana, India*					
Bhat et al. (2009)	Cross-sectional	Dahod district (mixed urban and rural), India	Chiranjeevi Scheme (vouchers for maternal health services)The programme was introduced in five districts in Gujarat, India, in 2005. Women with documentation that indicate eligibility can receive free maternity care services at participating private facilities. Facilities are reimbursed for every 100 women who they provide care to.	Vouchers for maternity care services	PN
De Costa et al. (2014)	Retrospective area study	Gujarat, India		Further research needed	FB
Ganguly et al. (2014)	Qualitative – interviews	Two districts in Gujarat, India		Vouchers for maternity care services, but with greater attention to developing trust between service providers and communities	Implementation
Jega (2007)	Qualitative – interviews	2 districts in Gujarat (mixed urban and rural), India		Vouchers for maternity care services, but with antenatal and postnatal care included, supply-side investment (to establish blood banks) and formal monitoring systems	Implementation
Mohanan et al. (2014)	Cross-sectional	Gujarat, India		Further research needed	ANC, FB, PN
*Voucher programme, Cambodia*					
Ir et al. (2010)	Qualitative – in-depth interviews and focus groups	3 districts in Kampong province, Cambodia	Voucher programme (vouchers for maternal health services)The programme was implemented in 22 districts between 2007 and 2010. Voucher distribution was universal in 14 districts and targeted in 8 districts. Eligible women were identified local volunteers and could use vouchers for free ANC, childbirth and PN/PP at public health facilities. Health centres were reimbursed for vouchers.	Vouchers for maternity care services alongside interventions to promote quality of care and to overcome non-financial barriers to demand	Implementation
Van de Poel et al. (2014)	Cross-sectional	Nationally representative sample, Cambodia		Vouchers for maternity care services, but universal distribution may be more effective than targeting	ANC, FB, C/I, PN
Vouchers for merit goods					
*Tanzanian National Voucher Scheme, Tanzania*					
Hanson et al. (2009)	Repeat cross-sectional	21 districts (mixed urban and rural), Tanzania	Tanzanian National Voucher Scheme (vouchers for merit goods)Women attending ANC were given a voucher that entitled them to a discounted insecticide-treated net (of any size) from an approved supplier. The programme began in 2004 and expanded globally over subsequent years.	Vouchers for merit goods	Ownership and use of insecticide-treated net
Koenker et al. (2013)	Qualitative – meetings, site visits and mathematical modelling	Five areas in Tanzania		Vouchers for merit goods, with voucher distribution in schools and at health clinics	Implementation
Mubyazi et al. (2010)	Qualitative – interviews and focus groups	Mkuranga and Mufindi districts (rural), Tanzania		No recommendation made	Implementation

ANC denotes antenatal care, SBA skilled birth attendance, FB births attended in healthcare facilities, PN postnatal care, PP postpartum care, C/I care-seeking for complications or illness in women and newborns, QoC quality of care, MM maternal mortality, PM perinatal mortality and NM neonatal mortality

- conditional cash transfers (Brazil’s Bolsa Familia [17, 18], El Salvador’s Comunidades Solidarias Rurales [19], Guatemala’s Mi Familia Progresa [20], Programa de Asignación Familia in Honduras [21], the Muthulakshmi Reddy Maternity Benefit Scheme in India [22, 23], Indonesia’s Program Keluarga Harapan [24–26], Mexico’s Oportunidades [27–34], and Plan de Atención Nacional a la Emergencia Social (PANES) in Uruguay [35]);
- unconditional cash transfers (Zambia’s Child Grant Programme [36]);
- short-term cash payments to offset costs (CHIMACA in China [37], India’s Janani Suraksha Yojana [38–74], the Safe Delivery Incentive Programme in Nepal [75–77] and the SURE-P programme in Nigeria [78]);
- vouchers for maternity care services (Bangladesh’s Maternal Health Voucher Scheme [79–84], a pilot programme in Bangladesh [85], a voucher programme in Cambodia [86, 87], the Chiranjeevi Yojana in India [88–92], Kenya’s Vouchers for Health programme [93–102], pilot programmes in Pakistan [103, 104], and the HealthyBaby vouchers [105, 106] and Mekerere University Voucher Scheme in Uganda [107, 108]), and vouchers for merit goods (the Tanzanian National Voucher Scheme [109–111]).

Programmes ranged from small-scale pilot voucher schemes in Pakistan and Bangladesh to large national programmes such as Janani Suraksha Yojana in India, Bangladesh’s Maternal Health Voucher Scheme and multiple conditional cash transfer programmes in Latin American countries. Programme design varied from those that were purely demand-side to those that included supply-side incentives such as output-based payments to service providers (for example many of the voucher programmes), or incentives for community-based workers (such as in India’s Janani Suraksha Yojana). Funding for programmes has come from national and state governments or from donor organisations such as the German Agency for International Cooperation (GIZ), the UK Department for International Development and the Bill and Melinda Gates Foundation.

The studies were generally of medium quality. Many of the quantitative studies were conducted early in the implementation of programmes and some made only limited efforts to account for confounding factors. Qualitative studies were often part of larger programme evaluations that focused primarily on quantitative outcomes and some of the articles reporting these findings lacked detailed descriptions of the methods used to collect and analyse data. Despite these limitations an overview of this literature does offer insights into the programmatic processes across a wide range of DSF initiatives and allows us to identify common features across programmes as well as some programme specific challenges.

### Stakeholder perspectives and experiences

Three groups of stakeholders in DSF programmes have been studied in most detail: women service users, community-based workers, and staff in health facilities. Documented experiences from each of these groups are reviewed below and relate to awareness of programmes, cultural attitudes, perceptions of maternity care services, reasons for using or joining programmes and the challenges faced during participation. Many findings reflect wider issues in healthcare systems, however this section focuses on DSF implementation, and findings have been disaggregated by type of DSF where possible.

#### Women who are potential DSF programme users

Target groups may be unaware of programme details [24, 25, 41, 46, 50, 58, 59, 61, 63, 64, 67, 71, 76, 77, 81, 85, 89, 91, 98, 101]. Women and their families may not realise that they are eligible for programmes (or incorrectly think that they are) and may not know which facilities they can use through the scheme [48, 63]. Remote areas may be less likely to receive promotional activities [93, 101]. Effective forms of awareness generation have included community dissemination of information [81], radio broadcasts [93], and networks of women’s groups [19, 28, 29, 77].

Social and cultural attitudes towards women play an important role in utility of vouchers as well as in use of services. Some women reported not being able to use a voucher because their husband did not want to be labelled as poor [98, 105], because they were expected to return to a family home elsewhere to give birth [105], or because nobody was available to accompany them to a participating hospital [63, 71, 85]. Of those who did travel to facility for birth care, many sought early discharge in order to return to look after children [52].

Perceptions of the quality of care and behaviour of providers were important for utilisation of health services, and therefore the implementation of DSF schemes. Prospective users were discouraged by reputations that facilities had long waiting times [81, 85], were poorly equipped and unclean [39, 49, 58, 61, 63, 65, 71, 80, 85, 92], or were places where one encountered disrespectful and abusive care [33, 44, 49, 50, 52, 54, 56, 61, 63, 81, 86, 94, 101, 105]. Modesty rules made some women reluctant to be treated by male doctors [58] or to visit midwives where it is considered inappropriate to let someone else see one’s genitals [25]. Other deterrents included fear of being subjected to unwanted procedures such as injections, surgical procedures and stitches [61, 71, 72] and of testing for HIV at a health facility and the attached stigma of HIV [98, 101]. Regular antenatal care visits may help women to become familiar and more relaxed with facilities and staff [52], and community visits by staff and women who were satisfied with their care could promote uptake [71, 73].

#### Community-based workers

Workers based in communities (including voucher distributors) can be important facilitators for DSF programmes. India’s accredited social health activists (ASHA) were found to be have played an important role in raising the awareness of Janani Suraksha Yojana and assisting women to obtain payments [38, 39, 52, 56, 58, 63, 64, 68–70, 72, 112], as did workers for Program Keluarga Harapan in Indonesia [24, 25]. Community health workers were an important source of information on DSF programmes in India [64, 88] and Tanzania [109], and voucher distributors performed a similar role in Kenya [99], Pakistan [103, 104] and Cambodia [87].

Community-based workers occupy a challenging position between communities, DSF programmes and health facilities. In communities, they can face criticism and accusations of theft if cash transfer payments are delayed [23], or if women go to a facility to give birth but do not receive payments they had been told about, or receive a smaller amount than expected [49, 56, 58, 65]. When visiting facilities, community-based workers who are reimbursed for facilitating DSF programmes may be asked for informal payments by facility staff [50, 56]. They may be used as go-betweens to request money from families on behalf of service providers and service providers have reportedly punished those who resist by refusing to register the facilitator’s subsequent service users, referring them to other facilities unnecessarily, or withholding DSF payments [44, 50, 56]. In some cases women have been reluctant to allow workers to accompany them to a facility because they feared money would be taken from them [48].

The potential of community-based workers to implement DSF programmes can also be undermined by geographically over-large operating areas that entail long distances for travel [25]. Some female workers were restricted by their family members in when they could accompany pregnant women to a facility [56]. Some workers’ usefulness was limited by their poor knowledge of programme details [53] and others reported difficulties in applying eligibility criteria because their guidelines were not clear [23]. Financial issues included resistance to their continued work for the programme from their families if their payment was delayed [56], and having to pay for food and transport when accompanying a women for antenatal care yet not receiving any reimbursement if the woman later gave birth at home [47, 56, 58]. Other difficulties include the risks of co-option of schemes by community members for personal gain. Positions as programme workers are sometimes regarded by communities as ‘lucrative’ compared to other forms of work [44], and there have been reports of recruitment processes being hijacked by local politicians and community leaders to appoint family members even though they were unlikely to want to perform the necessary duties [48, 65].

#### Staff in health facilities

Experiences among service providers and managers at participating health facilities seem to be mixed. In evaluations of voucher schemes, government and private providers report gaining skills and experience, making investments in infrastructure, being able to hire more staff, and reduced absenteeism among existing staff [86, 106, 108]. However, staff at some facilities in voucher schemes felt that increased user-load and administrative work was not adequately compensated by provider payments [79, 82, 106]. Some felt that schemes designed to target specific groups created problems and complained that they had been threatened by women who were excluded by means-testing criteria [81], or subjected to pressure from local politicians to distribute vouchers to ineligible women [81].

In short-term cash payment programmes, service providers at government facilities reported a significant increase in their workload exacerbating the existing inadequacies of services [40, 46, 50, 54, 59, 61]. Knowledge of schemes varied. Some government providers in India admitted that they knew nothing about Janani Suraksha Yojana other than that they needed to give payments to women who gave birth in the facility [54, 59]. One study reported that women were treated with hostility by facility staff when they sought cash assistance [54]. On the other hand, delayed or irregular reimbursements by programmes could lead to difficulties distributing money to women and expose the service providers as targets for criticism from families [48, 76].

In programmes designed to allow opt-in of private providers, respondents reported that they joined to help the poor to access health services [81, 90, 91] and conversely to increase business [81, 90]. In one study some said they felt coerced, fearing that they would otherwise be subjected to unwarranted but damaging investigations [90]. Private providers reported subsequently dropping out of voucher schemes because they felt ‘overwhelmed’ by the number of voucher users [93], or were unhappy with inadequate and delayed payments [108]. Some private providers in the Chiranjeevi Scheme in India reported that revenue had fallen because there were too few voucher users, their facility had gained an unwanted reputation as a place for poor people, and that pregnant women who used to pay fees to be attended there were now using the voucher programme [90]. Providers in the Tanzanian National Voucher Scheme for insecticide-treated nets stated that they enjoyed participating but that the programme required substantial investment of money and storage space to stock slow-selling nets, at the expense of other more popular items such as soap [110].

### Barriers and facilitators to successful implementation

Seven themes were identified regarding barriers and enabling factors for the implementation of DSF programmes: scope of the programme, supply-side capacity, contracting private providers, administrative processes and procedural considerations, information systems, fraudulent practices and their control, and sustainability issues.

### Scope of the programme

A common barrier found across many DSF programmes was that they were insufficient in their scope to overcome continued financial, social and geographical barriers to accessing services. Vouchers for maternity care services typically cover treatment costs but many do not include other important ‘demand-side’ costs such as transport, which can be prohibitive for households [79, 85, 88, 92, 96, 98–101, 104–106]. There is also the opportunity cost for women and their families of spending time away from their home and children [86, 103]. In contexts where pregnant women are asked to buy vouchers, for example the Vouchers for Health programme in Kenya, women may be deterred by the price of vouchers [96, 100]. Experiences with vouchers for merit goods in Tanzania have been similar. Women highlighted the cost of travel to obtain a voucher from health facilities, the cost of travelling to an approved shop to use the voucher and the remaining cost of the insecticide-treated net (which was only part-subsidised by a voucher) [109, 111].

Possible options to increase the accessibility of voucher services include, inclusion of travel costs within voucher entitlements [80, 81, 85, 100, 107], community distribution of vouchers to reduce travel to *obtain* a voucher and accreditation of additional facilities and providers to reduce travel to *make use* of a voucher [105]. For example, the Maternal Health Voucher Scheme in Bangladesh included short-term cash payments to facilitate uptake by offsetting costs of access [79].

In short-term payment programmes and in cash transfer schemes, payments may simply be too small or too late to offset out-of-pocket costs that include transport, tests, medicines and sutures [23, 25, 33, 37, 44, 46–49, 54, 55, 58–61, 66, 67, 77]. The size of payments may need to be increased periodically to keep pace with inflation and the Muthulakshmi Reddy Maternity Benefit Scheme in India has repeatedly increased the amount paid to women during its 30 year implementation period [22]. The programme offered 300 rupees to eligible women when launched in 1987 and now offers 12,000 rupees (approximately USD 190).

Provisions for onward referral in the case of an obstetric complication have often not been included in DSF schemes yet should be considered (see also sections on supply-side capacity and on procedural considerations below). Some families reported having to pay for treatment costs after they were referred to a non-participating facility [101], and others returned home when faced with additional expenditure [54, 66]. Bangladesh’s Maternal Health Voucher Scheme included ‘seed funds’ that could be used by health facilities for supply-side investments and to pay for emergency transport for voucher users [81].

Narrow eligibility criteria were highlighted as a barrier by studies of many DSF programmes. For example, schemes that exclude women who have more than a certain number of children are reported as unfair, difficult to enforce and completely counter-intuitive for programmes that aim to reduce maternal mortality [22, 76, 79, 82]. If a decision is made to design a targeted programme then use of locally appropriate poverty screening tools should be considered [94, 95, 98, 99, 101, 106]. Programmes in India have often used an existing system of ‘below poverty line’ cards however such programmes are constrained by any pitfalls in the existing system such as non-ownership of cards by those in need but lacking documentation, and leakage of cards to those least in need [88].

#### Supply-side capacity

The contextual evidence highlights that DSF cannot work well without adequate supply-side investment in public services and systems, and many of the findings in this sub-section are indicative of wider challenges in healthcare systems. Many evaluations of DSF programmes described problems at health facilities as a key barrier. Poor availability of medicines and other medical supplies – sometimes linked to bureaucratic procedures for procurement that discourage restocking [48] – meant that women in Bangladesh and India needed to make considerable personal expenditure [81, 82, 85]. Bed shortages meant many women were discharged within 24 h of giving birth, which may not allow sufficient time for post-partum care [46, 48]. In Indonesia’s Program Keluarga Harapan, cash transfers were contingent on using midwives however village midwife posts were reported to be unfilled in many areas [25]. Contracting private providers in voucher schemes does not remove the need for quality public sector provision as private providers often refer complicated cases to the public sector [105].

In India, where DSF schemes can be found in many states, there have been widespread reports of poor quality of care and improper practices, linked to increased workloads of staff in health facilities [40, 55, 60, 63]. It is important to monitor quality of care and adverse outcomes after childbirth, however DSF programmes were reported to lack adequate monitoring systems in this area [45, 46, 50] and women reported having no way to register and process their complaints at health facilities [50, 66]. Incidents included delays in starting treatment for women with serious conditions [66], chaotic delivery rooms [40], low utilisation of partographs [43], babies being left unmonitored in birth pans [44], physical abuse of women [44, 66], episiotomies conducted without permission and stitched without local anaesthesia [44], high incidence of oxytocin injections for labour induction [45], and pregnant women who have been diverted from a public to a private facility at the behest of an owner who worked on-call at the public facility [41].

Referrals from one facility to another are a key issue that may put the life of the woman and her baby at risk and are a cause of substantial expenditure for families who face additional costs for transport, food and accommodation [41, 45, 50, 53, 54, 66, 70, 76, 79]. Reported reasons for referral related to wider healthcare systems issues including absence of specialists at lower level facilities [42, 50, 59, 79], busy or absent doctors and midwives [49, 50, 58, 62, 66, 67], and a lack of functioning equipment for operations or blood transfusions (including faulty or stolen generators) [40, 48, 50, 65, 91]. In India, private practitioners in the Chiranjeevi Yojana were reluctant to provide care for clusters of women requiring care for complications due to the associated costs and the risk of adverse outcomes [90, 91].

There are two key enabling factors for programme designers and managers to support facilities and promote care-seeking. Firstly, designers need to realistically examine the capacity of local health systems to provide care to women who use DSF programmes. This needs to include emergency transport [62], round-the-clock opening hours [38, 60] and clearly stated guidelines for onward referrals [45, 50, 66, 69]. Procurement systems for essential medicines may need to be included within programme designs, at least until government procurement systems can be adequately strengthened [101].

Secondly, designers may consider how best to support participating facilities to maintain and improve the quality of care. Concurrent supply-side investments can improve working conditions for staff and expand service coverage, and ‘seed funds’ have reportedly been used to good effect in Bangladesh’s Maternal Health Voucher Scheme [81]. El Salvador’s Comunidades Solidarias Rurales and Guatemala’s Mi Familia Progresa were reported to have been implemented successfully alongside investments in the infrastructure and human resources of health facilities [19, 20], and similar supply-side investments have been important in India [38, 60] and Nigeria [78]. Another approach that has been advocated is to link provider payments to uptake of services [77, 87, 94, 107]. For programmes using government facilities (including if they are used for onward referrals), it is important that government facilities receive any linked payments and are able to invest them in infrastructure and human resources [93, 101, 105]

#### Contracting private providers

For programmes that are designed to include the opt-in of private providers, there is a risk that programmes will struggle to maintain sufficient number of participating providers if revenue from service users is considered to be inadequate [90, 91, 93, 101]. Providers and programme managers have reported that attempts to contract private providers to implement Janani Suraksha Yojana in some Indian states struggled due to low provider payments [42] and lack of interested providers that meet accreditation criteria [68].

Four particular issues have been documented for the contracting of private providers in voucher programmes. Firstly, providers may deliver differential treatment to voucher users compared to fee-paying pregnant women [93, 98]. Secondly, providers may seek to increase revenue by charging pregnant women for services that should be provided free according to the DSF scheme guidelines and agreements, including ultrasound scans, medicines and surgical care [88, 91, 92]. Thirdly, some providers may engage in the ‘skimming’ of voucher users requiring little intervention and referral of those requiring operations to avoid incurring costs [90, 91], or avoid the risk (and associated litigation) of being held responsible for any adverse outcomes [90]. Fourthly, providers may withdraw from a programme [89, 90, 93, 108].

Programme designers could consider how best to reimburse private facilities for childbirth. As noted above, private facilities may be reluctant to perform surgical interventions if reimbursements are the same regardless of level of intervention [90, 91], and programme officials in India suggested that provider contracts should include a clause stating that caesarean sections must be provided to any voucher user who needed one. Conversely, there are fears that differential reimbursement rates may provide an incentive for participating private facilities to perform high rates of caesarean sections, and that close monitoring may be an important deterrent [83].

#### Administrative processes and procedural considerations

Requirements for formal documents to be produced in order to prove eligibility should be carefully considered as in some cases the undocumented will be those most in need, including migrants, young and multiparous women [22, 41, 50, 53, 55, 58, 76, 94, 101]. In Kenya, for instance, young women were effectively excluded from a voucher scheme as the required government identification cards are only issued at 18 years of age [101]. It may be necessary to allow alternative forms of evidence (such as photos or letters signed by community leaders) [46], or to avoid restrictions and requirements for formal documents altogether [41, 46, 53].

For short-term cash payment programmes, rigid insistence on bureaucratic processes is a barrier to payment claims by women and some women did not travel to a facility for childbirth if they had heard reports other women had been denied money [48, 49]. The distribution of payments was considered to lack transparency [46, 58], and respondents reported being repeatedly sent away from facilities to obtain additional documents [41, 76]. Payments have been denied for reasons including because a seven-day claim period had passed, women were more than 12 weeks gestation at the time of registration, an official would not approve payments to women who gave birth before he took his post, a woman had given birth on the way to a facility and a woman was not accompanied by a community-based worker [41, 48, 68, 76].

It is important that payments to users and participating facilities in DSF programmes use streamlined and timely systems. For short-term cash payment programmes, women received payments as late as 12 months after giving birth [46], or not at all [76], and some families reported selling possessions or incurring debts to pay for transport and medicines for childbirth [47]. Service providers have suggested that such payments to women should be made before childbirth [71], however this may not be enough to overcome irregular disbursements of funding to local levels [41, 42, 58, 72].

The distribution of payments to users can be an administrative burden for officials and service providers, who may not fully understand the programme [58, 59]. Some service providers highlighted a need for more support to cope with paperwork [59, 67], while others had restricted payments to a specific desk at a certain time of day to cope with demand for payments [59]. Local officials suggested that community health workers who manage accounts and distribute payments should be trained accordingly [68, 69].

Payments to health facilities have reportedly been delayed due to bureaucratic procedures and a lack of understanding among facility staff regarding what supporting documentation must be submitted [82, 90, 93, 97, 101]. Stringent fraud detection systems may cause delays [106], but need to be balanced against the effects of fraud (see section on corruption). Providers can benefit from feedback mechanisms on how to make payment claims [93]. District officials reacted to unpredictability in funding by adopting first come, first served approaches, sharing smaller amounts among women, borrowing money from other sources, or using their own money [76].

#### Information systems

In targeted DSF programmes, information on target groups can help to inform locally appropriate poverty screening tools but may be difficult to obtain or use [94, 95, 98, 99, 101, 106]. Use of existing government systems for monitoring helps to reduce duplication [81, 82, 84], however these need to be functional – district officials in India and Nepal reported not having time, resources or guidelines to conduct monitoring visits [59, 76] and stated that they had to assume quality of care was good unless they heard otherwise [68]. An alternative approach advocated by studies in the review was for communities and non-governmental organisations to be supported to monitor quality of care, provision of free services and distribution of vouchers or cash transfers [45, 70]. Another suggested approach was to contract monitoring to an external organisation, although experiences with Kenya’s Vouchers for Health programme indicated that close scrutiny was needed to ensure that the contracted organisation performed monitoring processes as mandated [95, 101]. Such external oversight will also require additional financial outlay and may cause tensions within health services and administrations.

#### Fraudulent practices and their control

Fraudulent practices (by users, community-based workers and providers) have been documented as occurring in many DSF programmes. Local government officials may attempt to use programmes to get care for ineligible family members and friends [90], and officials in India and Nepal are reported to have embezzled programme funds [44, 76]. A recurring complaint across many short-term cash payment and voucher schemes is that families have been exposed to demands for informal payments by staff in health facilities [39, 44, 45, 47, 48, 50, 56, 60, 63, 77, 86, 93, 101]. In short-term cash payment programmes, staff have been known to deduct money before giving the scheduled payments [44, 46, 53, 59, 63], and indeed one study in India reported that around half of the amount due to be paid to women through Janani Suraksha Yojana was deducted by service providers [44]. Suggestions from officials include using pre-printed cheques and tracking these using online financial reporting systems [58, 70].

Community-based workers have also been known to apply informal charges to women and their families [44, 86, 93, 101]. Commission-based (rather than salaried) payments for such workers may have unintended consequences including workers placing inappropriate pressure on women to travel to health centres to give birth [53], and issuing vouchers to ineligible women in order to achieve the requisite numbers [93]. Initiatives taken by programme managers in Kenya to reduce dishonest behaviour included putting up posters with the true cost of vouchers on market days to prevent informal fees, and switching from commission-based to stipend-based payments for voucher distributors [93].

Some studies have emphasised the importance of strong monitoring systems [106, 108]. If false claims are reported sufficient resources need to be allocated so that they can be adequately followed-up [76]. Similarly, it is important to be able to remove providers from a programme if they are found to be engaged in malpractice [106], although in practice this may be difficult in districts where there are few providers or little interest in joining a programme.

#### Sustainability issues

The experience with DSF programmes ranges from cash transfers that have been in operation for almost 30 years, to voucher programmes that have only been implemented as short pilot programmes. Thorough planning and political support seem important for the sustainability of DSF programmes and programme designers need to ensure that all organisations involved in the programme have sufficient capacity to perform mandated tasks [59, 78, 81, 95]. Research has emphasised the importance of good communication between the different levels and different organisations [68, 77, 93]. Planning may take several years as appropriate systems and expertise are developed, and this may be an expensive process [24, 78]. Data should be monitored during implementation to adjust programme design as needed [93, 95]. Local government officials, community leaders and community-based workers can play an important role raising awareness of programmes [55, 81, 84, 101], however regular communication is needed as turnover of officials and poor communication between levels may lead to confusion and distribution of misinformation [79, 81, 84, 106].

Policy champions in donor and governmental organisations proved helpful to ensure political and financial support for programmes in Kenya and Uganda [93, 101, 106]. In Kenya the Vouchers for Health programme reportedly received support from national policy-makers because it was seen as a useful model to prepare for a national social health insurance programme [93]. However, programmes may also become subject to specific political interests. In Nepal, the national government reportedly forced programme roll-out before planning was complete and created tensions with state governments [76].

Ministry of Health engagement is often sought in donor-initiated programmes [84, 101], but programmes may become an administrative burden for under-resourced departments and facilities [81, 84]. In Kenya, a planned transfer of ownership to the Ministry of Health was repeatedly delayed and eventually required the assistance of a contracted consultancy firm [93].

## Discussion

This analysis has highlighted a series of well-documented challenges for the implementation of DSF schemes in maternal and newborn health. These include issues of programme scope (in terms of programme eligibility, size and timing of payments and voucher entitlements), wider problems in healthcare systems (including inadequate infrastructure and human resources, lack of medicines and problems with corruption) and the population’s awareness and perceptions of programmes and health services. A recent systematic review concluded that despite evaluations spanning 15 years of implementation, DSF programmes have yet to demonstrate positive impact of programmes on quality of care or maternal and newborn health outcomes [14]. That finding may reflect insufficient attention during programme design and implementation to improving the quality of care being provided and to conditions of access to comprehensive emergency care [113]. Research highlighted in our analysis indicates that the implementation of DSF programmes may reinforce existing healthcare system problems including poor quality of care, demands for informal fees and the systematic exclusion of vulnerable groups. Vouchers for maternity care services are often proposed as a means to improve quality of care however experiences indicate private providers may find reimbursement rates to be unattractive and engage in practices such as providing differential quality of care or ‘skimming’ programme users who require minimal intervention. Taken together, these findings suggest a need for greater attention to issues of implementation in DSF programmes and to the context in which they are to be introduced. They indicate serious concerns for the use of DSF as a stand-alone interventions in maternal and newborn health in low- and middle-income countries, and raise questions regarding which (if any) maternal and newborn healthcare services are suited to modalities of DSF.

Where DSF programmes have improved care-seeking, the programmes have tended to include (or be accompanied by) additional investment in health facilities or staff, while those without such investment have struggled (see Tables 2 and 3). Other important factors that have enabled DSF programmes to improve care-seeking have included appropriate payment size and timing for short-term cash payments and cash transfer programmes, and an adequate package of entitlements (including transport costs) in voucher schemes. When well-supported and -supervised, community-based workers, leaders and women’s groups have been important facilitators for programmes as they have raised awareness of programme details and helped to counter negative perceptions of programmes.

**Table 2:** Summary of key findings from quantitative studies on short-term cash payments and cash transfers

Programme	Effect on care-seeking outcomes^a^	Key findings from synthesis of factors influencing implementation	
		Enablers	Barriers
*Conditional cash transfers*			
Comunidades Solidarias Rurales, El Salvador	Increased skilled birth attendance and facility births. No effect on antenatal care and postnatal care	-Awareness generation during monthly meetings [19] -Concurrent investments in health facility infrastructure and recruitment of health workers [19] -Payments made to women (not their husbands) [19]	None stated
Mi Familia Progresa, Guatemala	Increased antenatal care. No effect on facility births	-Concurrent investments in health facility infrastructure and recruitment of health workers [20]	None stated
Programa de Asignación Familia, Honduras	Increased antenatal care. No effect on postnatal care	-Conditionalities to submit paperwork at health facilities [21]	-Poor awareness among women of programme conditionalities [21]
Muthulakshmi Reddy Maternity Benefit Scheme, India	Associated with use of public facilities for antenatal care and childbirth	-Increased total amount of payments [22]	-Delays in receipt of money for women [22] -Overly bureaucratic process for determining eligibility [22] -Eligibility criteria that restrict payments to women for her first or second live birth [22]
Program Keluarga Harapan, Indonesia	Increased antenatal and postnatal care. Mixed picture of positive and no effect on skilled birth attendance. No effect on facility births	-Awareness generation by community-based workers [24]	-Poor awareness of the programme among target groups [24] -Delays in receipt of money for women [24] -Failure to implement verification systems to penalise households that do not meet conditionalities [24] -Poor availability of midwives [26] -High start-up costs [24]
Oportunidades, Mexico	Mixed picture of positive and no effect on skilled birth attendance. No effect on antenatal care	-Awareness generation during monthly meetings [28, 29]	-Perceived poor behaviour of staff at participating hospitals [33] -Attitudes towards formal maternity care services of family members who do not attend monthly meetings [34] -Distance to participating facilities [33] -Cost of travel to health facilities [33] -Lack of concurrent investment in health facilities [28, 29]
Plan de Atención Nacional a la Emergencia Social (PANES), Uruguay	Increased antenatal care. No effect on skilled attendance at birth.	-Conditionalities for antenatal care were not enforced [35]	-Conditionalities for antenatal care were not enforced [35]
Unconditional cash transfers			
Child Grant Programme, Zambia	No effect on skilled birth attendance or antenatal care	None stated	-Lack of concurrent investment in health facilities [36]
*Short-term cash payments*			
CHIMACA programme, China	No effect on antenatal care or postnatal care	None stated	-Payment too small [37] -Overly difficult process for claiming money [37]
Janani Suraksha Yojana, India	Increased skilled birth attendance and facility births. Mixed picture of positive, negative and no effect on antenatal care and postnatal care	-Awareness generation by community-based workers [38, 39] -Round-the-clock opening of health facilities [38, 60] -Emergency transport programmes [62] -Accreditation of remote health facilities to reduce travel distances [55] -Active involvement of state and district officials [55] -Detailed implementation plans [38]	-Poor awareness of the programme among target groups [63] -Distance and lack of transport to participating facilities [55, 60] -Payments not made until after childbirth, thereby reducing incentive for antenatal care [55, 62] -Delays in receipt of money for women [39, 51, 55, 60, 63] -Demands for additional or informal payments [39, 60, 63] -Perceived poor quality of care at participating facilities [39] -Overly bureaucratic process for determining eligibility [55] -Inappropriate proxy measures of poverty, such as caste [38] -Women who travel to another area to give birth [51] -Delays in recruitment of community-based workers [39] -Poor awareness of the existence of community-based workers [63] -Delays in payments for community-based workers [55] -Increased workloads and reduced quality of care at participating health facilities [55, 62, 63] -Lack of trained midwives [62] -Existence of a similar programme – the National Maternity Benefit Scheme [55]
Safe Delivery Incentive Programme, Nepal	Increased antenatal care. Mixed picture of positive or no effect on skilled birth attendance and facility births	-Awareness generation by women’s groups in communities [77] -Lack of geographical barriers in the study district [77] -Universal eligibility [77] -Output-based reimbursements for providers [77]	-Poor awareness of the programme among target groups [77] -Delays in receipt of money for women [77] -Payments not sufficient to cover treatment costs [77] -Demands for additional or informal payments [77] -Overly difficult process for claiming money [77] -Confusion amongst health workers and officials regarding eligibility criteria, sharing of health worker incentives and payment mechanisms for women [77]
SURE-P programme, Nigeria	No effect on skilled birth attendance or antenatal care	-Prompt payments to pregnant women [78] -Defined roles and contracts for local banks and for organisations that will develop information systems [78] -Concurrent programmes to expand availability of maternity care services [78]	-Increased workload at participating health facilities [78] -High start-up costs including research, advocacy, development of information systems, recruitment of workers for data collection and the logistics and security of payments to pregnant women [78]

^a^See systematic review for further details of effect on care-seeking outcomes [14]

**Table 3:** Summary of key findings from quantitative studies on vouchers

Programme	Effect on care-seeking outcomes ^a^	Key findings from synthesis of factors influencing implementation	
		Enablers	Barriers
*Vouchers for maternity care services*			
Maternal Health Voucher Scheme, Bangladesh	Increased skilled birth attendance, facility births, antenatal care and postnatal care	-Activities by community workers and local leaders to raise awareness of the programme [81] -Inclusion of transport costs [80, 81] -‘Seed’ funds for participating facilities to promote investment in services and capacity [81]	-Perceived poor quality of care at participating facilities [81] -Pressure from local politicians to distribute vouchers to ineligible women [81]
Pilot vouchers, Bangladesh	Increased skilled birth attendance, facility births, antenatal care and postnatal care	-Inclusion of transport and medicine costs [85]	-Poor awareness of the programme among target groups [85] -Perceived poor quality of care at participating facilities [85]
Voucher programme, Cambodia	Increased skilled birth attendance and postnatal care. No effect on antenatal care.	-Awareness generation by voucher distributors [87] -Output-based reimbursements for providers [87]	None stated
Chiranjeevi Yojana, India	No effect on skilled birth attendance, antenatal care and postnatal care	-Community health workers provided information on the programme [88] -Use of an existing government system (‘below poverty line’ cards) as a targeting mechanism [88]	-Poor awareness of the programme among target groups [89] -Distance to participating facilities in rural areas [88] -Cost of transportation [92] -Demands for additional or informal payments [88, 92] -Providers not reimbursed for postnatal care [88] -Provider attrition in urban areas [89] -Waning political commitment [89]
Vouchers for Health, Kenya	Increased skilled birth attendance and facility births. No effect on antenatal care and postnatal care	-Awareness generation by voucher distributors and previous service users [99] -Locally appropriate tool for targeting pregnant women from low-income households [99] -Output-based reimbursements for providers [94]	-Perceived poor behaviour of staff at participating hospitals [94] -Distance and lack of transport to participating facilities [96, 100] -Cost of travel to health facilities [99, 100] -Cost of purchasing vouchers [96, 100] -Overly bureaucratic process for determining eligibility [94] -Delays in contract signing and voucher printing [99]
Pilot vouchers, Pakistan	Increased facility births. Mixed picture of positive and no effect on antenatal and postnatal care	-Awareness generation by voucher distributors [103, 104]	-Many women left the facility within 24 h after giving birth as there was no one to look after their homes and children [103] -Distance to participating facilities [104]
Makerere University Voucher Scheme, Uganda	Increased facility births	-Inclusion of transport costs [107] -Output-based reimbursements for providers [107]	None stated
HealthyBaby vouchers, Uganda	Increased facility births, antenatal care and postnatal care	-Locally appropriate tool for targeting pregnant women from low-income households [106]	-Turnover of staff in the Ministry of Health [106] -Cost of travel to health facilities [106] -Procedural burden of fraud detection system [106] -Inclusion (based on geographical needs) of facilities that did not meet minimum quality requirements [106]
*Vouchers for merit goods*			
Tanzanian National Voucher Scheme	Increased use of insecticide-treated nets	-Awareness generation by service providers [109]	-Distribution of vouchers during antenatal care visits misses women who do not seek formal antenatal care [109] -Cost of purchasing insecticide-treated net (even at a reduced rate) [109]

^a^See systematic review for further details of effect on care-seeking outcomes [14]

The longest-running DSF programmes included in this review have been cash transfer schemes that were introduced by national or state governments as part of wider social welfare programmes. Programmes since the launch of the Millennium Development Goals have tended to focus more narrowly on specific maternal health services with the aim to improve their coverage on indicators such as skilled attendants at birth or facility births. Most evaluations have shown quantitative improvements in coverage rates [14]. However, many have relied on funding from donors, and some were implemented for only short pilot periods. Studies have highlighted the high start-up costs of DSF programmes [24, 78], and those running in parallel to welfare systems are likely to have continued high overhead costs. Policy-makers need to consider whether DSF programmes that involve cash payments or vouchers are likely to be an optimal use of resources, or whether increased supply-side investment would be equally effective.

### Limitations of the review

The scope of literature included systematic review may have been limited by use of English search terms and English language databases. Findings were incorporated from a range of studies, including some that were of low quality, in order to gain insights for implementation from a wide range of contexts. The most common methodological weaknesses of studies related to the length of time for follow-up after programme initiation (quantitative studies), and failure to address to role of the researcher on data generation and analysis (qualitative studies). By including studies with such weaknesses, there is a risk that findings reported in this review over-emphasise short-term factors affecting implementation, and that findings are subject to unknown biases based on the value systems and social positioning of researchers.

## Conclusions

After quite widespread implementation and considerable policy enthusiasm in some quarters, evaluations have shed light on the importance of a number of detailed design and implementation issues as outlined above. The synthesis of findings reveals a complex picture of experiences DSF programmes in maternal and newborn health. While they indicate that cash payments and vouchers can be successful in improving service utilisation rates at least in the short term in an array of contexts, there are frequent concerns about inclusion criteria or distribution mechanisms that effectively exclude migrants, young and multiparous women, about staff charging informal fees once at the facilities, and about the struggle to maintain quality of care under greater demand. Unsurprisingly, the programmes that have successfully promoted uptake of specific maternity care services using cash or voucher incentives, such as Nepal’s Safe Delivery Incentive Programme and Bangladesh’s Maternal Health Voucher Scheme, are those which were carefully designed with adequate scope (in terms of programme eligibility, payment size and timing, or services and goods to which they provide entitlement) to properly address maternal and newborn health aims, were well-supported in communities and/or which operated within effective healthcare systems.

Research is still needed in a number of areas and the opportunity to update our original systematic review has reminded us of the narrow range of issues and indicators included in many evaluations of DSF programmes. Review papers on this topic are at risk of tunnel vision as a result. It is our considered view that after almost 15 years of evaluating DSF schemes there are some questions about the DSF approach which are important but rarely posed. For example, we found no evaluations that attempted to gauge the experience of coercion when birth in a health facility becomes a conditionality for women to receive state welfare payments. There is comparatively little evidence on the implementation of unconditional cash transfers as part of maternal and neonatal health programmes, despite growing interest in these within the international development community. Similarly, comparative research on alternative forms of financing, such as health equity funds, would provide useful insights. Furthermore, research on policy processes and the reasons for introducing DSF schemes rather than efforts to remove user fees or improve supply-side quality of care could also help to generate understanding of the role of these initiatives, how they become sustainable and where they fit (or do not fit) with plans to achieve equitable universal health coverage.

## Abbreviations

ASHA: Accredited social health activists
DSF: Demand-side financing
EPHPP: Effective Public Health Practice Project
GIZ: German Agency for International Cooperation
HIV: Human immunodeficiency virus
PANES: Plan de Atención Nacional a la Emergencia Social
SURE: Supporting the Use of Research Evidence framework
USD: US dollars
